# Indoleamine 2,3-dioxygenase and ischemic heart disease: a Mendelian Randomization study

**DOI:** 10.1038/s41598-019-44819-7

**Published:** 2019-06-11

**Authors:** Mengyu Li, Man Ki Kwok, Shirley Siu Ming Fong, Catherine Mary Schooling

**Affiliations:** 1 School of Public Health, Li Ka Shing Faculty of Medicine, The University of Hong Kong, Hong Kong, China; 2 City University of New York, Graduate School of Public Health and Health Policy, New York, NY USA

**Keywords:** Molecular medicine, Cardiology, Genetics

## Abstract

Tryptophan is an essential amino acid. Indoleamine 2,3-dioxygenase (IDO), the rate-limiting enzyme in the tryptophan-kynurenine pathway, is positively associated with cardiac events, and may be relevant to cancer. We used Mendelian Randomization to obtain unconfounded estimates of the association of IDO1 with ischemic heart disease (IHD), ischemic stroke and their risk factors, all-cancer, cancer of the prostate, lung and bronchus, and breast. We obtained genetic instruments independently and strongly (p-value < 5 × 10^−8^) predicting plasma IDO1 from a proteome genome-wide association study (GWAS), and applied them to consortia GWAS of the outcomes, including the UK Biobank SOFT CAD GWAS (cases < = 76 014, non-cases < = 264 785) for IHD. Estimates were obtained using inverse variance weighting; with MR-Egger, weighted median and MR-PRESSO as sensitivity analyses. IDO1 was inversely associated with IHD (odds ratio (OR) 0.96 per standard deviation, 95% confidence interval (CI) 0.93 to 1.00, p-value = 0.04), diabetes (OR 0.91, 95% CI 0.85 to 0.97) and prostate cancer (OR 0.96, 95% CI 0.93 to 0.99) with a directionally consistent estimate for stroke (OR 0.98, 95% CI 0.95 to 1.02) but not with blood pressure, or the other cancers considered. IDO1 might be a potential therapeutic target for IHD, diabetes and prostate cancer.

## Introduction

Tryptophan is an essential amino acid in humans that must be obtained from the diet, and is widely consumed in protein-based foods^1^, such as milk^2^, soy products and egg white^3^. More than 90% of dietary tryptophan, is catabolized via the kynurenine pathway^4^. Indoleamine 2,3-dioxygenase (IDO), one of the rate-limiting enzymes in the first step of the tryptophan-kynurenine pathway, may control the pathway under pathological conditions^5^ and have immunomodulatory^5,6^ and signaling functions^7^. IDO has two isoforms, IDO1 and IDO2, of which IDO1 is the major one controlling tryptophan degradation^8^. Kynurenine further degrades via different pathways (Supplementary Figure 1). Kynurenine--oxoglutarate transaminase (KAT), which has 4 isoforms (1–4), is responsible for degrading kynurenine to kynurenic acid^9^.

Despite considerable progress in the prevention of non-communicable diseases, such as cardiovascular diseases, ischemic heart disease (IHD) is still the leading cause of mortality and morbidity worldwide^10^. Investigating new prevention and treatment approaches is crucial. The tryptophan-kynurenine pathway is thought to be involved in the pathology of IHD and its risk factors including diabetes, obesity and immune-related diseases^11^. In most observational studies, IDO activity (measured as the kynurenine/tryptophan ratio) is positively associated with early atherosclerosis^12^ and IHD^13^, and correspondingly is inversely associated with prognosis in stroke patients^14^. However, in most animal experiments, inhibition of IDO or IDO deficiency in mice leads to early atherosclerosis and plaque instability^15,16^, although one study showed an inverse association of IDO1 deficiency with the risk of atherosclerosis^17^. IDO induction also exerts protective effects against atherosclerosis in mice^18–20^. These effects could be due to IDO1-dependent effects on lipid metabolism and inflammation^16^. More holistically, IHD is increasingly being considered within the well-established evolutionary theory that growth and reproduction trade-off against longevity^21^, gonadotropin-releasing hormone increases risk of IHD^22^. IDO activation might result in lower serotonin in the brain^23^, which may decrease gonadotropin-releasing hormone synthesis^24^ and secretion^25^. Notably, IDO1 inhibitors are under development as a cancer treatment, raising the possibility of re-purposing. However, no randomized controlled trials of IDO or IDO inhibitors to prevent or treat IHD have been conducted. Similarly, effects of KAT3 on IHD have rarely been studied, but its catabolite kynurenic acid might support the cardiovascular system by protecting the endothelium during hyperhomocysteinemia^26^.

Inconsistencies between observational studies and animal experiments may be due to the difficulty of eliminating confounding and distinguishing between causes and biomarkers of disease in observational studies, as well as differences in metabolism between humans and mice. In this situation, Mendelian Randomization (MR), as instrumental variable analysis with genetic instruments, may provide insight. Since the randomization of genetic makeup at conception is in some ways similar to the randomization process in randomized controlled trials^27^, MR provides unconfounded estimates of causal effects. Given tryptophan is a common dietary item^28^, we conducted an MR study to investigate the association of IDO1, and KAT3 for completeness, with IHD, ischemic stroke and their risk factors, including type 2 diabetes and blood pressure, using the largest available genetic consortia. Moreover, IDO inhibitors are being considered in cancer given their immunomodulatory property that prevents immune escape of cancer cells^29^ but a recent trial cast doubt on their effects on cancer^30^. For completeness we also assessed the association of IDO1 and KAT3 with all-cancer and common cancers including prostate, lung and bronchus, and breast cancer.

## Results

The F statistic for the single nucleotide polymorphisms (SNPs) available in the analyses predicting plasma IDO1 at 5 × 10^−8^ was 155.3, while in sensitivity analyses at 5 × 10^−6^ it was 72.3. The F statistic for the SNPs predicting plasma KAT3 available in the analyses at 5 × 10^−8^ was 68.7, while in sensitivity analyses at 5 × 10^−6^ it was 29.2. This study had at least 80% power at 5% α to detect an odds ratio of 0.97 for IHD per standard deviation of IDO1. The results of power calculations for all the outcomes are available in Supplementary Table 1. For the SNPs available on the GTEx Portal, we found the tissues where the instruments were expressed were consistent with IDO1 and KAT3 expression. For example, the instruments predicting IDO1 are expressed in blood, esophagus and skin (Supplementary Table 2), in which IDO1 protein is expressed.

### Associations of genetically predicted IDO1 and KAT3 with IHD and stroke

Table 1 shows genetically instrumented IDO1 was inversely associated with IHD but not ischemic stroke using inverse variance weighting (IVW) with directionally consistent estimates using weighted median (WM) at 5 × 10^−8^. MR-Egger did not show potentially pleiotropic effects. Sensitivity analyses at 5 × 10^−6^ gave similar results. Table 1 also shows that genetically instrumented KAT3 was not clearly associated with IHD or ischemic stroke using IVW at 5 × 10^−8^. However, in sensitivity analysis at 5 × 10^−6^ KAT3 was inversely associated with IHD but not with ischemic stroke using IVW, with a directionally consistent WM estimate. After removing 2 potentially pleiotropic SNPs related to systolic blood pressure and fat mass, identified from PhenoScanner, the association was not evident using IVW or any other method.

**Table 1:** Estimates of the effect of indoleamine 2,3-dioxygenase 1 (IDO1) protein and kynurenine--oxoglutarate transaminase 3 (KAT3) protein on ischemic heart disease (IHD) and ischemic stroke using Mendelian Randomization with different methods.

Outcome	Exposure (SD)	SNPs	Method	OR	95%CI	P value	Cochran’s Q statistic (p-value)	MR-Egger	
								Intercept p value	I^2^
IHD	IDO1	3^a^	IVW	0.96	0.93, 1.00	0.04	5.68 (0.06)	0.44	93.0%
			MR-Egger	1.05	0.83, 1.32	0.68			
			WM	0.96	0.92, 1.00	0.06			
		8	IVW	0.96	0.92, 1.00	0.03	10.96 (0.14)	0.25	93.9%
			MR-Egger	1.00	0.92, 1.10	0.95			
			WM	0.96	0.91, 1.00	0.07			
			MR-PRESSO	0.96	0.92, 1.00	0.03			
	KAT3	2^b^	IVW	0.95	0.89, 1.01	0.10	3.49 (0.06)		
		15	IVW	0.96	0.93, 1.00	0.04	18.08 (0.20)	0.06	39.4%
			MR-Egger	1.02	0.95, 1.10	0.52			
			WM	0.98	0.94, 1.03	0.50			
			MR-PRESSO	0.96	0.93, 1.00	0.04			
		13*	IVW	0.97	0.94, 1.01	0.16	14.79 (0.25)	0.07	36.5%
			MR-Egger	1.03	0.96, 1.10	0.43			
			WM	0.99	0.94, 1.04	0.63			
			MR-PRESSO	0.97	0.94, 1.01	0.17			
ischemic stroke	IDO1	3^a^	IVW	0.98	0.95, 1.02	0.30	4.55 (0.10)	0.96	93.6%
			MR-Egger	0.99	0.76, 1.29	0.93			
			WM	0.96	0.92, 1.00	0.08			
		8	IVW	0.98	0.94, 1.03	0.44	11.63 (0.11)	0.78	94.9%
			MR-Egger	0.97	0.88, 1.08	0.58			
			WM	0.96	0.92, 1.01	0.08			
			MR-PRESSO	0.98	0.94, 1.03	0.45			
	KAT3	2^b^	IVW	0.94	0.88, 1.00	0.06	0.23 (0.63)		
		11	IVW	0.99	0.95, 1.03	0.73	13.39 (0.20)	0.47	50.9%
			MR-Egger	1.03	0.93, 1.14	0.61			
			WM	0.99	0.94, 1.04	0.61			
			MR-PRESSO	0.99	0.95, 1.03	0.74			
		9*	IVW	1.00	0.95, 1.05	0.90	12.69 (0.12)	0.50	52.9%
			MR-Egger	1.03	0.92, 1.15	0.58			
			WM	1.01	0.96, 1.07	0.67			
			MR-PRESSO	1.00	0.95, 1.05	0.91			

*Exclusion of 1 SNP (rs9787133) related to systolic blood pressure, and 1 SNP (rs7500458) related to fat mass. ^a^Not enough instrumental variables for MR-PRESSO. ^b^Not enough instrumental variables for MR-Egger, WM and MR-PRESSO.

### Associations of genetically predicted IDO1 and KAT3 with type 2 diabetes

Table 2 shows that genetically instrumented IDO1 was inversely associated with type 2 diabetes using IVW with WM giving similar results at 5 × 10^−8^. MR-Egger did not show potentially pleiotropic effects. Sensitivity analyses at 5 × 10^−6^ gave similar results. Table 2 also shows that genetically instrumented KAT3 was inversely associated with type 2 diabetes using IVW at 5 × 10^−8^. However, at 5 × 10^−6^ the associations were not evident using IVW or any other method.

**Table 2:** Estimates of the effect of indoleamine 2,3-dioxygenase 1 (IDO1) and kynurenine--oxoglutarate transaminase 3 (KAT3) proteins on type 2 diabetes (T2DM) using Mendelian Randomization with different methods.

Outcome	Exposure (SD)	SNPs	Method	OR	95%CI	P value	Cochran’s Q statistic (p-value)	MR-Egger	
								Intercept p value	I^2^
T2DM	IDO1	3^a^	IVW	0.91	0.85, 0.97	0.003	0.86 (0.65)	0.64	90.7%
			MR-Egger	0.87	0.70, 1.07	0.18			
			WM	0.91	0.85, 0.97	0.01			
		8	IVW	0.92	0.87, 0.97	0.002	2.75 (0.91)	0.31	91.8%
			MR-Egger	0.87	0.76, 0.98	0.03			
			WM	0.92	0.86, 0.98	0.01			
			MR-PRESSO	0.92	0.89, 0.95	<0.001			
	KAT3	2^b^	IVW	0.87	0.78, 0.96	0.01	1.11 (0.29)		
		15	IVW	1.00	0.95, 1.05	0.97	13.42 (0.49)	0.46	22.9%
			MR-Egger	0.97	0.88, 1.07	0.51			
			WM	1.03	0.96, 1.10	0.40			
			MR-PRESSO	1.00	0.95, 1.05	0.98			

^a^Not enough instrumental variables for MR-PRESSO. ^b^Not enough instrumental variables for MR-Egger, WM and MR-PRESSO.

### Associations of genetically predicted IDO1 and KAT3 with systolic blood pressure and diastolic blood pressure

Table 3 shows genetically instrumented IDO1 was not clearly associated with systolic or diastolic blood pressure using IVW or any other method. At 5 × 10^−8^, KAT3 was inversely associated with systolic blood pressure but not with diastolic blood pressure using IVW, but in sensitivity analyses at 5 × 10^−6^ these associations were not evident.

**Table 3:** Estimates of the effect of indoleamine 2,3-dioxygenase 1 (IDO1) and kynurenine--oxoglutarate transaminase 3 (KAT3) proteins on systolic blood pressure (sbp) and diastolic blood pressure (dbp) using Mendelian Randomization with different methods.

Outcome (SD)	Exposure (SD)	SNPs	Method	beta	95%CI	P value	Cochran’s Q statistic (p-value)	MR-Egger	
								Intercept p value	I^2^
sbp	IDO1	3^a^	IVW	0.00	−0.01, 0.01	0.70	4.78 (0.09)	0.03	92.9%
			MR-Egger	0.03	0.00, 0.06	0.05			
			WM	0.00	−0.01, 0.01	0.69			
		8	IVW	0.00	−0.01, 0.01	0.52	6.26 (0.51)	0.16	94.5%
			MR-Egger	0.01	−0.01, 0.03	0.33			
			WM	0.00	−0.01, 0.01	0.71			
			MR-PRESSO	0.00	−0.01, 0.00	0.51			
	KAT3	2^b^	IVW	−0.02	−0.04, −0.01	0.01	20.74 (<0.01)		
		15	IVW	−0.01	−0.02, 0.01	0.30	39.98 (<0.01)	0.18	38.6%
			MR-Egger	0.01	−0.02, 0.04	0.50			
			WM	0.00	−0.01, 0.01	0.85			
			MR-PRESSO (corrected)	0.00	−0.01, 0.01	0.98			
		14*	IVW	0.00	−0.01, 0.01	0.97	16.38 (0.23)	0.25	34.1%
			MR-Egger	0.01	−0.01, 0.03	0.33			
			WM	0.00	−0.01, 0.02	0.77			
			MR-PRESSO	0.00	−0.01, 0.01	0.97			
dbp	IDO1	3^a^	IVW	0.00	−0.01, 0.01	0.54	9.05 (0.01)	0.06	92.9%
			MR-Egger	0.04	0.00, 0.09	0.06			
			WM	0.01	0.00, 0.02	0.21			
		8	IVW	0.00	−0.01, 0.01	0.89	12.65 (0.08)	0.04	94.5%
			MR-Egger	0.02	0.00, 0.04	0.06			
			WM	0.01	−0.01, 0.02	0.30			
			MR-PRESSO	0.00	−0.01, 0.01	0.90			
	KAT3	2^b^	IVW	0.00	−0.02, 0.01	0.70	4.62 (0.03)		
		15	IVW	0.01	−0.01, 0.02	0.37	22.08 (0.08)	0.71	38.6%
			MR-Egger	0.00	−0.02, 0.03	0.94			
			WM	0.01	−0.01, 0.02	0.22			
			MR-PRESSO	0.01	−0.01, 0.02	0.38			

*Exclusion of 1 SNP (rs9787133) related to systolic blood pressure. ^a^Not enough instrumental variables for MR-PRESSO. ^b^Not enough instrumental variables for MR-Egger, WM and MR-PRESSO.

### Associations of genetically predicted IDO1 and KAT3 with cancers

Tables 4 and 5 show that genetically instrumented IDO1 was inversely associated with prostate cancer using IVW and WM with directionally consistent estimates in MR-Egger but not with all-cancer, lung and bronchus or breast cancer at 5 × 10^−8^. KAT3 was not clearly associated with any-cancer, prostate, lung and bronchus or breast cancer using IVW or any other method at 5 × 10^−8^. Sensitivity analyses at 5 × 10^−6^ gave similar results.

**Table 4:** Estimates of the effect of indoleamine 2,3-dioxygenase 1 (IDO1) and kynurenine--oxoglutarate transaminase 3 (KAT3) protein on all cancers using Mendelian Randomization with different methods.

Outcome	Exposure (SD)	SNPs	Method	Proba-bility	95%CI	P value	Cochran’s Q statistic (p-value)	MR-Egger	
								Intercept p value	I^2^
cancer	IDO1	3^a^	IVW	0.00	0.00, 0.00	0.86	1.79 (0.41)	0.77	92.9%
			MR-Egger	0.00	−0.01, 0.01	0.81			
			WM	0.00	0.00, 0.00	0.91			
		8	IVW	0.00	0.00, 0.00	0.63	7.19 (0.41)	0.34	94.5%
			MR-Egger	0.00	0.00, 0.01	0.51			
			WM	0.00	0.00, 0.00	0.63			
			MR-PRESSO	0.00	0.00, 0.00	0.65			
	KAT3	2^b^	IVW	0.00	−0.01, 0.00	0.91	0.91 (0.34)		
		15	IVW	0.00	0.00, 0.00	0.69	15.42 (0.35)	0.43	38.7%
			MR-Egger	0.00	−0.01, 0.00	0.38			
			WM	0.00	0.00, 0.00	0.67			
			MR-PRESSO	0.00	0.00, 0.00	0.70			

^a^Not enough instrumental variables for MR-PRESSO. ^b^Not enough instrumental variables for MR-Egger, WM and MR-PRESSO.

**Table 5:** Estimates of the effect of indoleamine 2,3-dioxygenase 1 (IDO1) protein and kynurenine--oxoglutarate transaminase 3 (KAT3) protein on prostate cancer, lung and bronchus cancer and breast cancer using Mendelian Randomization with different methods.

Outcome	Exposure (SD)	SNPs	Method	OR	95%CI	P value	Cochran’s Q statistic (p-value)	MR-Egger	
								Intercept p value	I^2^
prostate cancer	IDO1	3^a^	IVW	0.96	0.93, 0.99	0.01	4.43 (0.11)	0.38	93.6%
			MR-Egger	0.89	0.74, 1.06	0.19			
			WM	0.96	0.92, 1.00	0.04			
		8	IVW	0.96	0.93, 0.99	0.01	8.25 (0.31)	0.44	94.4%
			MR-Egger	0.94	0.87, 1.01	0.08			
			WM	0.96	0.92, 1.00	0.03			
			MR-PRESSO	0.96	0.93, 0.99	0.01			
	KAT3	2^b^	IVW	0.98	0.92, 1.03	0.42	4.26 (0.04)		
		15	IVW	1.00	0.95, 1.04	0.83	28.88 (0.01)	0.85	36.4%
			MR-Egger	0.99	0.90, 1.08	0.79			
			WM	1.00	0.95, 1.05	0.95			
			MR-PRESSO	1.00	0.95, 1.04	0.84			
lung and bronchus cancer	IDO1	3^a^	IVW	1.04	0.93, 1.17	0.48	0.25 (0.88)	0.81	92.9%
			MR-Egger	0.99	0.66, 1.51	0.98			
			WM	1.04	0.92, 1.18	0.52			
		8	IVW	1.00	0.90, 1.11	0.94	5.83 (0.56)	0.11	94.5%
			MR-Egger	1.18	0.94, 1.50	0.16			
			WM	1.03	0.91, 1.17	0.66			
			MR-PRESSO	1.00	0.91, 1.10	0.94			
	KAT3	2^b^	IVW	1.00	0.81, 1.24	0.97	0.31 (0.58)		
		15	IVW	1.09	0.96, 1.22	0.18	14.36 (0.42)	0.66	37.3%
			MR-Egger	1.03	0.79, 1.34	0.82			
			WM	1.05	0.89, 1.24	0.54			
			MR-PRESSO	1.09	0.96, 1.22	0.18			
breast cancer	IDO1	3^a^	IVW	0.99	0.97, 1.02	0.54	1.48 (0.48)	0.26	93.3%
			MR-Egger	0.94	0.87, 1.03	0.21			
			WM	0.99	0.96, 1.02	0.52			
		8	IVW	0.99	0.97, 1.01	0.37	7.55 (0.37)	0.80	94.3%
			MR-Egger	0.98	0.93, 1.04	0.56			
			WM	0.98	0.95, 1.01	0.21			
			MR-PRESSO	0.99	0.97, 1.01	0.38			
	KAT3	2^b^	IVW	1.03	0.98, 1.08	0.19	12.62 (<0.01)		
		15	IVW	1.00	0.97, 1.04	0.89	26.80 (0.02)	0.54	33.4%
			MR-Egger	0.98	0.91, 1.06	0.63			
			WM	0.99	0.95, 1.03	0.51			
			MR-PRESSO (corrected)	0.99	0.96, 1.01	0.29			

^a^Not enough instrumental variables for MR-PRESSO. ^b^Not enough instrumental variables for MR-Egger, WM and MR-PRESSO.

## Discussion

Consistent with previous animal experiments^15,16^ suggesting IDO is protective against atherosclerosis, this MR study showed that plasma IDO1 protein was inversely associated with IHD. We also showed that IDO1 and KAT3 were inversely associated with type 2 diabetes which has rarely been studied previously. We could not rule out the possibility that KAT3 was inversely associated with IHD and systolic blood pressure. We also found IDO1 inversely associated with prostate cancer but not other cancers considered.

An abnormal kynurenine pathway has been observed in atherosclerosis^11^, which is important in the development of IHD and is also an important therapeutic target to prevent IHD. In mice IDO inhibition by 1-methyl tryptophan (an IDO inhibitor) enhanced vascular inflammation by immune dysregulation, and thus increased atherosclerotic lesions^16^. IDO deficiency in mice accelerated early atherosclerosis by dysregulation of cytokine IL-10^15^. These experiments indicate that IDO1 could be protective against atherosclerosis possibly by an immune-inflammatory response.

In this MR study, we provide genetic validation of the potential role of IDO1 in IHD, by showing that life-long increased plasma IDO1 was inversely associated with risk of developing IHD. Although the association was not significant for ischemic stroke possibly because of lack of power and selection bias, the direction of the estimate was consistent with that of IHD, which gives some validation of our findings for IHD. However, this is not consistent with most observational studies in older people^12,13^ or in patients with cardiac events^14,31^, which show IDO activity (kynurenine/tryptophan ratio) positively associated with coronary events and cardiovascular risk factors. However, the kynurenine/tryptophan ratio is not a direct measure of IDO1. On the one hand, it may mainly reflect the enzymatic function of IDO1 or the effects of its catabolite kynurenine more than the complicated function of IDO1. On the other hand, not only IDO1 but also IDO2 and tryptophan 2,3-dioxygenase can affect this ratio^11^. In addition, studies of a fatal disease in older people are open to selection bias, which can generate reversed estimates^32^, as are studies in patients, as well as to unmeasured confounding. In addition, IDO can be induced by inflammation related cytokines^7,33^; the associations between IDO activity and intima-media thickness did not hold after adjustment for traditional risk factors^12,34^. As such, IHD might increase IDO activity as a negative feedback loop to protect against over-reaction by the immune system. However, we could not conduct an adequately powered bi-directional MR study because of the relatively small sample size of the genome-wide association study (GWAS) for IDO1.

Unexpectedly, we also found that IDO1 was protective against type 2 diabetes. Type 2 diabetes causes IHD^35^. Preventing diabetes might be a pathway by which IDO1 lowers the risk of IHD. Previous studies of IDO1 and type 2 diabetes are limited, although some have suggested diabetes up-regulates the tryptophan-kynurenine pathway^36,37^. Given IDO1 has complex and multiple functionality^7,38^, its effects on type 2 diabetes as well as the underlying mechanism, such as via insulin given products of the kynurenine pathway inhibit the synthesis and activity of insulin^39^, needs further investigation. Unexpectedly, we also found KAT3 might be protective against diabetes and high systolic blood pressure but not IHD. These associations were not evident in sensitivity analysis at 5 × 10^−6^ so these findings need further confirmation. How these findings relate to our recent findings that other specific kynurenine related factors, i.e., aspartate but not glutamate, protect against IHD^40^, also needs to be considered.

Given IDO’s immunosuppressive function, IDO inhibitors have been considered for cancer treatment, and have passed phase 1^41^ and phase 2^42^ trials. However, recently a pivotal trial of an IDO inhibitor failed to accomplish its primary and secondary outcome in treating melanoma, casting doubt on its efficacy for treating cancer^30^. In this MR study, we also found that IDO1 was not associated with most cancers considered, consistent with this latest trial. However, we found IDO1 was protective against prostate cancer, which is rarely studied, although one study suggested it as a biomarker for prostate cancer^43^.

This MR study made full use of the largest publicly available GWAS giving adequate power, while avoided the confounding that may affect conventional observational studies. However, some limitations still exist. First, in MR studies, weak instruments could bias the association towards the null. We used genetic predictors associated with exposures at genome-wide significance (5 × 10^−8^), and used a less stringent cut off 5 × 10^−6^ for sensitivity analysis. One SNP rs7010461 (IDO1 gene) is functionally associated with IDO1 with a small p-value of 2.45 × 10^−20^, and was not associated with IHD, diabetes, systolic and diastolic blood pressure or cancer at genome-wide significance. Larger F statistics indicate lower probability of weak instrument bias with a conventional threshold of 10^44^, and the F statistics were all larger than 10 in this study. Second, we were not able to assess the association of genetic predictors with a wide range of unknown confounders, but none of the SNPs used was associated with known confounders including age of completing education, current tobacco smoking, alcohol intake frequency, walking frequency and prescription of anti-depressants at Bonferroni corrected significance. Third, we could not rule out the possibility of horizontal pleiotropy. However, we used different methods with different assumptions to detect potential horizontal pleiotropy statistically, including MR-PRESSO. We also checked for known pleiotropy and removed such SNPs in a sensitivity analysis. Fourth, although MR is less susceptible to confounding, it can be confounded by ethnicity, i.e., population stratification. We used GWAS with participants mainly of European ancestry with genomic control^10,45–51^. Fifth, we were not able to confirm whether associations varied by baseline levels of IDO1. Sixth, since IDO1 could affect the cardiovascular system via several different pathways, the associations could be non-linear but we assumed it was linear. Seventh, we did not pre-specify exhaustively what we considered pleiotropic effects, potentially adding some subjectivity to the assessment of known pleiotropy. Eighth, we did multiple testing for different outcomes here, which may increase type I error, i.e., false positives^52^. However, in our hypothesis IHD was the primary outcome in this study, and other outcomes such as traditional risk factors of IHD were mainly used for further investigation of potential pathways. Ninth, IDO and KAT have different isoforms, but here we only studied the effects of IDO1 and KAT3 due to data availability. However, IDO1 is the major isoform responsible for tryptophan degradation to kynurenine^8^, while KAT3 shares high similarity with KAT1 and may also relate to glutamine and histidine^53^. Finally, MR estimates are vulnerable to selection bias in the underlying GWAS, here CARDIoGRAMplusC4D and DIAGRAM. However, CARDIoGRAMplusC4D shows the expected associations for PCSK9 and HMGCR genetic variants^54^, while GWAS of less-fatal conditions such as diabetes are less vulnerable to selection bias.

## Conclusions

Consistent with animal experiments and evolutionary biology, we obtained genetic validation of plasma IDO1 protein as protecting against IHD, with directionally consistent results for stroke. Unexpectedly, both IDO1 and KAT3 were inversely associated with type 2 diabetes, suggesting the kynurenine pathway also plays a role in development of diabetes. Consistent with evolutionary biology, we also found IDO1 was also protective against prostate cancer. Given the complex function of IDO1 and wide consumption of the essential amino acid tryptophan in our diets, IDO1 could be a potential target for IHD, diabetes and prostate cancer prevention worthy of further investigation. Intervening on IDO1 such as modifying iron intake^55^, might help to prevent IHD^56^ and diabetes.

## Methods

### Genetic associations with exposures: IDO1 and KAT3

SNPs strongly associated with IDO1 and KAT3 proteins (p-value < 5 × 10^−8^) were obtained from the most recent plasma proteome GWAS in 3301 healthy participants of European descent (mean age 43.7 years, 51.1% men), adjusted for age, sex, duration between blood draw and processing and the first three principal components of ancestry from multi-dimensional scaling^45^. SNPs associated with these enzymes at a less stringent significance level (5 × 10^−6^) were used in sensitivity analyses. We selected uncorrelated SNPs (r^2^ < 0.05) with a smaller p-value based on the 1000 Genomes catalog^57^. SNPs that were not available for an outcome were replaced by proxies. Potentially palindromic SNPs (A/T or C/G) were also replaced by proxies where available, because SNP-specific effect allele frequency was not given for the proteome study.

Four independent SNPs (r^2^ < 0.05) predicted IDO1 at p-value < 5 × 10^−8^ but rs8110965 was potentially palindromic and had no proxy (r^2^ > 0.6) (Supplementary Tables 3 and 4). Nine independent SNPs predicted IDO1 at p-value < 5 × 10^−6^, rs75781101 was proxied by rs17817660 (r^2^ = 1.0) for all outcomes but rs8110965 had no proxy (r^2^ > 0.6) (Supplementary Tables 3 and 4).

Only two independent SNPs predicted KAT3 at p-value < 5 × 10^−8^, rs9787133 was proxied by rs7539070 (r^2^ = 0.96) for all outcomes (Supplementary Tables 5 and 6). Sixteen independent SNPs predicted KAT3 at p-value < 5 × 10^−6^. Among these SNPs, three SNPs were replaced by proxies for IHD, ischemic stroke and diabetes, two for blood pressure, all-cancer, prostate cancer and breast cancer, and four for lung and bronchus cancer, but no proxy for some SNPs, such as rs12935125, was available (Supplementary Tables 5 and 6).

### Genetic associations with the outcomes: IHD, its risk factors including type 2 diabetes and blood pressure, as well as cancers

Genetic associations with IHD were obtained from the most up to date and largest GWAS in the CARDIoGRAMplusC4D consortium, which is a meta-analysis of the UK Biobank SOFT CAD GWAS with studies from the CARDIoGRAMplusC4D consortium^10^. The UK Biobank SOFT CAD GWAS was adjusted for array and the first five principal components^10^. The final GWAS included up to 76 014 cases and 264 785 non-cases mainly of European descent, with double-genomic-control. Genetic associations with ischemic stroke were obtained from the MEGASTROKE consortium, which meta-analysed 29 GWASs mainly of Europeans. The final study included 60341 ischemic stroke cases and 454450 controls^51^. Genetic associations with type 2 diabetes were from the Stage 1 Summary statistics of the DIAGRAM 1000 Genomes GWAS meta-analysis which combined 18 GWASs. The GWAS included 26 676 cases and 132 532 controls mainly of European ancestry, adjusted for age, sex and principal components of ancestry^46^. Genetic associations with automated-reading systolic and diastolic blood pressure (standard deviation) were from the Neale Lab UK Biobank summary statistics (n = 340 159 for systolic blood pressure, n = 340 162 for diastolic blood pressure) mainly of people of white British ancestry, adjusted for the first 20 principal components, age, age^2^, sex, age*sex and age^2^*sex^47^. The UK Biobank is a large cohort comprising ~500 000 individuals from the UK, aged 40–69 years at recruitment^47^. Genetic associations with self-reported diagnosis of any cancer were from the same source of summary statistics similarly adjusted (cases = 28 509, controls = 331 472)^47^. Genetic associations with prostate cancer were obtained from the largest available meta-analyses of summary statistics in the PRACTICAL consortium. The GWAS includes 79148 prostate cancer cases and 61106 controls of European ancestry, adjusted for principal components^48^. Genetic associations with lung and bronchus cancer were from UK-Biobank Single Variant Association Analysis Results using SAIGE, which includes 2101 cases and 406226 controls of white British ancestry adjusted for the first four principal components, sex, and birth year^49^. Genetic associations with breast cancer were obtained from the BCAC consortium, which includes 122977 cases and 105974 controls of European ancestry, adjusted for principle components and sometimes additionally adjusted for country and study^50^. A comprehensive description of the sources of the genetic associations is presented in Supplementary Table 7.

### Statistical analysis

We calculated the F statistics for all the instruments combined using an approximation, i.e., average of the estimated SNP specific F statistics (square of beta for SNP to exposure/square of its variance)^58^. We identified any genetic instruments associated at Bonferroni-corrected significance with potential confounders including age of completing education, current tobacco smoking, alcohol intake frequency, walking frequency and prescription of anti-depressants in the UK Biobank summary statistics^47^. Whether the genetic instruments are linked to the outcomes directly rather than through the exposures (pleiotropic effects) was assessed by checking their known phenotypes obtained from comprehensive curated genotype to phenotype cross-references, i.e., PhenoScanner (http://www.phenoscanner.medschl.cam.ac.uk/) and the GWAS Catalog (https://www.ebi.ac.uk/gwas/). Power was calculated for each outcome^59^, on the basis that the sample size for instrumental variable analysis is the sample size for regression of exposure on outcome divided by the r^2^ for instrument on exposure^60^. To further validate the SNPs we used, we checked the genotype tissue expression data using the GTEx Portal (https://gtexportal.org/home/) where available. Since IDO1 is expressed by dendritic cells^61^ which are present in the skin^62^, lungs^61^, stomach^63^, intestines^61^, blood and lymph nodes^61^, and KAT3 is expressed widely including in the skin, gastrointestinal tract and lungs^64,65^, we checked whether the instruments were expressed in these tissues.

Estimates of the associations of IDO1 and KAT3 with IHD, ischemic stroke, type 2 diabetes, blood pressure (systolic and diastolic) and cancers were obtained by meta-analysing the SNP-specific Wald estimates (ratio of SNP on outcome to SNP on exposure) using IVW with multiplicative random effects for 4+ SNPs and fixed effects for <4 SNPs^66^. IVW with random effects assumes balanced pleiotropy (i.e., the mean of the horizontal pleiotropic effect is zero so does not bias the estimate)^67^ while IVW with fixed effects assumes all SNPs are valid instruments^66^. We used sensitivity analyses with different assumptions to check the robustness of the IVW estimates. WM gives reliable estimates when over 50% of the weight is contributed by valid instruments^68^. MR-Egger and MR-PRESSO (Mendelian randomization pleiotropy residual sum and outlier) are used to test directional (unbalanced) pleiotropy. MR-Egger allows invalid instruments and is robust to pleiotropy assuming the instrument strength is independent of direct effect^58^. A non-null MR-Egger intercept indicates directional pleiotropy and a less reliable IVW estimate^58^. MR-Egger has low power and does not identify pleiotropic SNPs. MR-PRESSO detects potentially pleiotropic SNPs based on the residual sum of squares and corrects for them by removing statistically significant outliers^69^. We reported the corrected MR-PRESSO estimate if given otherwise the raw estimate. All statistical analyses were conducted using R version 3.5.0 (The R Foundation for Statistical Computing, Vienna, Austria). The MR-Base (TwoSampleMR) R package was used for selecting uncorrelated SNPs and aligning effect alleles, MendelianRandomization and the MRPRESSO R packages were used to obtain estimates. All the data were publicly available and thus no ethical approval was required for this study.

### Ethics approval and consent to participant

All the data were publicly available and no individual data were used. Therefore, no ethical approval and consent was required for this study.

## Supplementary information

Supplementary materials

## Data Availability

All data generated or analysed during this study are included in this article (and its Supplementary Files).
